# Characterization and therapeutic potential of newly isolated bacteriophages against *Staphylococcus* species in bovine mastitis

**DOI:** 10.1128/jvi.01901-24

**Published:** 2025-02-14

**Authors:** Jae-hyun Cho, Gyu Min Lee, Seyoung Ko, Youngju Kim, Donghyuk Kim

**Affiliations:** 1 Optipharm Inc., Cheongju-si, Chungcheongbuk-do, Republic of Korea; 2 School of Energy and Chemical Engineering, Ulsan National Institute of Science and Technology (UNIST) 684828 , Ulsan, Republic of Korea; Michigan State University, East Lansing, Michigan, USA

**Keywords:** bovine mastitis, bacteriophage, *Staphylococcus*, *Herelleviridae*, protein structure analysis

## Abstract

Bovine mastitis, primarily caused by *Staphylococcus aureus*, significantly affects the dairy industry by reducing milk production and quality. The rise of antibiotic-resistant bacteria has prompted the need for alternative treatments. The three newly isolated bacteriophages, OPT-SA02, OPT-SC01, and OPT-SX11, were isolated from chicken fecal and sewage samples in South Korea. These bacteriophages were characterized via physiological and genomic analyses, identifying their therapeutic potential against *S. aureus*-induced mastitis. The bacteriophages were identified as members of the *Herelleviridae* family, exhibiting stability across broad pH (2–12) and temperature (37–70°C) ranges, as well as strong antibacterial activity at low multiplicity of infection (MOI) levels. Genomic analysis revealed that the conservation of lysis-related genes (holin and endolysin) is responsible for their lytic capabilities. Additionally, protein structural predictions revealed multi-domain structures in their endolysins, enhancing their lytic potential. These findings suggest that OPT-SA02, OPT-SC01, and OPT-SX11 show significant promise as alternative treatments for bovine mastitis.

**IMPORTANCE:**

Bovine mastitis, caused by pathogens such as *Staphylococcus aureus* and *Staphylococcus xylosus*, remains a major challenge in dairy farming, leading to significant economic losses and reduced milk quality. The increasing prevalence of antibiotic-resistant strains further complicates treatment, emphasizing the need for alternative strategies. This study identifies three newly isolated bacteriophages with effective antibacterial activity against these pathogens and provides comprehensive genomic and structural insights into their mechanisms. Genomic characterization revealed conserved lytic cassettes and genetic diversity within related bacteriophages, offering a deeper understanding of their evolutionary relationships and potential applications. Furthermore, protein structure analysis of the endolysin derived from these bacteriophages identified multi-domain architectures with preserved catalytic cores, underscoring their lytic efficacy against bacterial cell walls. These findings advance the understanding of the genetic and structural mechanisms of bacteriophage-mediated lysis and highlight their potential as sustainable tools for managing bovine mastitis and improving milk quality in dairy farming.

## INTRODUCTION

Gram-positive bovine mastitis, which is primarily caused by staphylococci, such as *Staphylococcus aureus* and coagulase-negative staphylococci (CoNS), leads to substantial economic losses due to reduced milk production and quality, affecting large-scale dairy industries globally (1–5). The virulence factors of *S. aureus*, such as hemolysin, toxic shock syndrome toxin-1, leukocidin, fibronectin-binding proteins (FnbA and FnbB), encapsulation, microabscess formation, and the promotion of biofilm development by clumping factors (ClfA and ClfB), can result in chronic infections and promote the occurrence of antibiotic resistance (6–8).

Antibiotics are routinely used to treat intramammary infections caused by *Staphylococcus* species (9). However, the frequent use of beta-lactam antibiotics, particularly penicillinase-resistant types such as methicillin and oxacillin, has led to the emergence of methicillin-resistant *Staphylococcus aureus* (MRSA) and CoNS, which poses persistent challenges for both animal and human health due to limited treatment options (10–12). Given the rapid emergence of antibiotic-resistant strains, such as MRSA and CoNS, traditional antibiotic treatments are becoming increasingly ineffective. This highlights an urgent need for alternative therapeutic strategies, including lysins, antibodies, probiotics, immune stimulation, and phage therapy, to counteract these resistant pathogens (13, 14). Recent advancements in bacteriophage-derived and engineered endolysins have demonstrated significant potential as alternative antimicrobials for combating bovine mastitis caused by *S. aureus* and CoNS (15–17). These endolysins target bacterial cell walls with high specificity, bypassing traditional mechanisms of antibiotic resistance.

With increasing restrictions on antibiotic use in livestock production, interest in alternative antimicrobial strategies has increased (18–20). The use of bacteriophages, which are viruses that specifically infect antibiotic-resistant bacteria (21–24), has emerged as a targeted approach to control bacterial pathogens in animal feed additives and therapeutics (25–30). Lytic phages, in particular, kill bacterial cells through lysis (23, 24). The use of phage cocktails has demonstrated effectiveness in inhibiting bacterial growth and reducing *S. aureus* in bovine mastitis, specifically targeting mastitis infections in livestock (31–34). By combining different phages, phage cocktails enhance the range of susceptible bacterial strains while retaining host specificity.

Unlike antibiotics, bacteriophages exhibit narrow host specificity, which is particularly advantageous in livestock production, as it minimizes disruption to beneficial microorganisms. In livestock, *Staphylococcus* species are considered part of the normal microbiota, functioning as commensals, but under specific conditions, they can transition into pathogens (35, 36). For instance, CoNS, such as *Staphylococcus chromogenes*, are frequently isolated from the bovine udder and are considered as part of the natural udder microbiome (37). Some CoNS strains have even demonstrated protective effects against major mastitis pathogens like *S. aureus* and *Streptococcus uberis* by inhibiting bacterial growth and stimulating the immune response. However, certain species, such as *S. aureus*, are primary causative agents of bovine mastitis and other infections in cattle, while specific *Staphylococcus* strains can lead to localized infections, such as comb necrosis, in poultry (36). In this context, bacteriophages, with their ability to selectively target pathogenic *Staphylococcus* species while minimally disrupting beneficial microorganisms, represent a promising approach for therapeutic applications in livestock.

However, to effectively utilize these bacteriophages, it is essential to thoroughly characterize their antimicrobial activity and stability under various conditions (38). Bioinformatics analyses, including genetic analysis, structural prediction, and exploration of functional domains, help in understanding the genetic diversity of bacteriophages and their evolutionary relationships (39). Functional analysis of lytic proteins, such as endolysins, further elucidates the potential applications of isolated bacteriophages. Structural characterization of endolysins that degrade bacterial cell walls is particularly important for understanding their efficacy and stability (40). These analyses require knowledge of the three-dimensional structure of proteins, and recent advancements in structural biology, particularly with deep learning-based structure prediction algorithms, have enabled the reliable prediction of target protein structures (41, 42).

In this study, we present a comprehensive phenotypic and genomic characterization of three bacteriophages, OPT-SA02, OPT-SC01, and OPT-SX11. The host range, growth characteristics, antimicrobial activity, and stability of these bacteriophages are evaluated under various stress conditions, with the aim of specifically targeting *S. aureus*-related mastitis. Their potential as therapeutic agents for bovine mastitis was assessed by examining the ability to inhibit bacterial growth in milk. Additionally, we conducted bioinformatics-based characterization to elucidate the genetic composition and structural insights into their lytic proteins, facilitating the development of effective therapeutics and management strategies for mastitis.

## MATERIALS AND METHODS

### Bacterial strains and growth conditions

*Staphylococcus aureus* and *Staphylococcus xylosus* were selected as the host bacteria to isolate bacteriophages (Tables 1 and 2). Specifically, the host strains used for each bacteriophage were as follows: OPT-SA02 utilized *S. aureus* SAU18, and both OPT-SX11 and OPT-SC01 utilized *S. xylosus* KCCM 40887. These host strains were routinely cultivated in tryptic soy broth (TSB, BD, Germany) with shaking at 37°C for 18 hours. Other bacteria were also cultured with shaking in TSB media at 37°C for 18 hours.

**TABLE 1:** Susceptibility against isolated clinical strains

Species	Susceptibility (no. of susceptible bacteria/no. of tested bacteria)		
	OPT-SA02	OPT-SC01	OPT-SX11
*Staphylococcus aureus*	99% (99/100)	98% (98/100)	100% (100/100)
Methicillin-resistant *Staphylococcus aureus* (MRSA)	100% (7/7)	100% (7/7)	100% (7/7)
Coagulase-negative staphylococci (CNS)			
*S. borealis*	100% (3/3)	100% (3/3)	100% (3/3)
*S. chromogenes*	100% (3/3)	100% (3/3)	100% (3/3)
*S. epidermidis*	100% (3/3)	33% (1/3)	100% (3/3)
*S. haemolyticus*	100% (3/3)	33% (1/3)	100% (3/3)
*S. saprophyticus*	100% (3/3)	100% (3/3)	100% (3/3)
*S. sciuri*	100% (3/3)	0% (0/3)	33% (1/3)
*S. simulans*	100% (3/3)	100% (3/3)	67% (2/3)
*S. xylosus*	100% (3/3)	100% (3/3)	100% (3/3)
Total	99% (130/131)	93% (122/131)	98% (128/131)

**TABLE 2:** Host specificity determination of the isolated bacteriophages^*a*^

Species	Strain ID	Susceptibility		
		OPT-SA02	OPT-SC01	OPT-SX11
Gram-positive bacteria				
*Bacillus cereus*	ATCC 11778	-	-	-
*Streptococcus iniae*	KCTC 3657	-	-	-
*Enterococcus faecalis*	K07 EFL-12–001	-	-	-
*Enterococcus faecium*	K14 EFM-13–001	-	-	-
*Streptococcus uberis*	KVCC-BA0001861	-	-	-
*Streptococcus dysgalactiae*	KVCC-BA0001419	-	-	-
Gram-negative bacteria				
*Salmonella* Typhimurium		-	-	-
*Salmonella* Enteritidis		-	-	-
*Escherichia coli*		-	-	-
*Klebsiella pneumoniae*		-	-	-

^*a*^ -, no susceptibility.

### Isolation and purification of bacteriophages

The bacteriophages were isolated from chicken fecal and sewage samples in Chungcheongbuk-do, South Korea, via a previously described protocol (43). Briefly, the samples were filtered through a 0.2 µm pore size membrane filter. The filtrate, 10× TSB, and host bacterial culture were mixed and incubated at 37°C for 18 hours. After centrifugation at 6,000 rpm for 20 minutes, the supernatant was filtered through a 0.2 µm membrane filter. TSB containing 0.6% agar was mixed with 200 µL of host bacteria and poured onto tryptic soy agar (TSA) plates. The filtrate was spotted on these bacterial lawns, and lytic activity was observed after 24 hours of incubation at 37°C.

To purify the bacteriophages, the soft agar overlay method was used (44). The bacteriophage suspension was diluted with sodium chloride-magnesium sulfate (SM) buffer (100 mM NaCl, 10 mM MgSO_4_, 50 mM Tris–HCl [pH 7.5]) by 10-fold dilutions. A single plaque was picked and suspended in 1 mL of SM buffer at room temperature for 6 hours. The suspension (100 µL) was mixed with bacterial culture (200 µL) and TSA with 0.6% agar (12 mL), and then poured onto TSA plates containing 2% agar. After 18 hours of incubation at 37°C, the plaques were extracted by adding 15 mL of SM buffer and stirring at 100 rpm for 6 hours. The suspension was centrifuged at 3,500 rpm and 4°C, and filtered through a 0.22 µm membrane filter. All experiments were performed at least in triplicate to obtain the bacteriophages.

### Transmission electron microscopy (TEM)

A suspension of bacteriophages at a concentration of over 1.0 × 10^9^ plaque-forming units (PFU)/mL was placed onto glow-discharged carbon-coated copper grids. After allowing the sample to absorb for 2 minutes, excess liquid was removed using Whatman paper. The sample was then treated with a 2% (wt/vol) uranyl acetate (UrAc) solution for 1 minute to stain the bacteriophages. After blotting off excess UrAc, observations were made using a Tecnai G2 Spirit Twin microscope (FEI, USA) at an acceleration voltage of 120 kV.

### Host specificity determination for bacteriophages

The host range of bacteriophages was evaluated using 100 methicillin-susceptible *Staphylococcus aureus* (MSSA) strains, 7 MRSA strains, 24 CoNS, as well as 6 gram-positive bacteria and 4 gram-negative bacteria. A total of 131 *Staphylococcus* species were obtained from the Animal and Plant Quarantine Agency in South Korea (QIA), all of which were isolated from dairy farms. Specifically, 96 *S*. *aureus* and 23 CoNS strains were isolated from cows with mastitis, while the remaining 11 *S*. *aureus* and 1 CoNS strain were isolated from general dairy farm environments.

Additional gram-positive bacteria included *Bacillus cereus* ATCC 11778, *Streptococcus iniae* KCTC 3657, *Streptococcus uberis* KVCC-BA0001861, *Streptococcus dysgalactiae* KVCC-BA0001419, *Enterococcus faecalis*, and *Enterococcus faecium*, while the gram-negative bacteria included *Salmonella Typhimurium*, *Salmonella Enteritidis*, *Escherichia coli*, and *Klebsiella pneumoniae*. The host range of the bacteriophages was tested using the soft agar overlay method, as described in “Isolation and purification of bacteriophages,” above. Lytic activity was indicated by clear zones, demonstrating bacteriophage activity against the tested bacteria.

### Temperature and pH stability of bacteriophages

Heat and pH stability tests of OPT-SA02, OPT-SC01, and OPT-SX11 were performed according to a previous protocol (43). To determine phage stability under various temperature conditions, OPT-SA02, OPT-SC01, and OPT-SX11 at a final concentration of approximately 10^9^ PFU/mL were incubated at 37°C, 40°C, 50°C, 60°C, and 70°C for 1 hour. Phage titers were measured at 0, 10, 30, and 60 minutes. After incubation for 18 hours, the phage titers were determined via the soft agar overlay method (44). To determine phage stability under various pH conditions, acetic acid and sodium acetate buffer were used at pH 2–6, phosphate buffer at pH 7–11, and Tris–HCl buffer at pH 8–11. The pH of the buffers was adjusted with 1 M HCl or 1 M NaOH. Ten microliters of phage suspension (with a final concentration of approximately 10^9^ PFU/mL) was added to 990 µL of pH buffer and was incubated at room temperature for 1 hour. After treatment, the phage titers were determined using the soft agar overlay method.

### One-step growth curve

A one-step growth curve experiment to determine the burst size and latent period was conducted as described previously, with some modifications (45). Briefly, 10 µL of bacteriophage suspension (approximately 1.0 × 10^9^ PFU/mL) was added to 10 mL of exponential phase culture of *S. aureus* or *S. xylosus* to obtain a multiplicity of infection (MOI) of 0.01. The mixture was allowed to adsorb to the host strain for 10 minutes at 37°C. After phage adsorption, 100 µL of the mixture was added to 20 mL of fresh TSB medium. Samples were taken at 10-min intervals for 120 minutes, after which the bacteriophage titer was determined using the soft agar overlay method. All experiments were performed in duplicate to ensure reproducibility of the results.

### Bactericidal effects of the bacteriophages

To assess the inhibitory effect of phages on *S. aureus* or *S. xylosus*, approximately 100 µL of host bacteria reaching optical density at 600 nm (OD600) of 1.0 was transferred into a sterile tube and mixed with 100 µL of bacteriophage suspension at different MOI levels: 1, 0.1, and 0.01. The control group consisted of bacterial cultures without phages. The samples were incubated with shaking at 37°C, and the OD600 was measured at 1-hour intervals for 7 hours via a spectrophotometer (Infinite M200PRO, TECAN, Switzerland) to monitor phage activity. All measurements were performed in triplicate.

### Activity in ultra-high temperature (UHT)-treated whole milk

To evaluate the inhibitory effects of bacteriophages on *S. aureus* or *S. xylosus* in milk, 500 µL of host bacteria (10^8^ CFU/mL) was added individually to 50 mL of UHT-treated whole milk (Mleko UHT Kocham Mleko/I love milk 3,5%, Mlekovita Co., Poland; product code 5900512984759) supplemented with 500 µL of bacteriophages (10^8^ PFU/mL). The control group included only bacterial cultures without bacteriophages. The mixture was incubated at 37°C for 0, 2, 6, and 24 hours. The viable cell counts of the injected bacteria were determined using the spread plate method. This involved observing the growth of colonies on TSA plates in the control and bacteriophage-treated groups. Specifically, the mixture was serially diluted 10-fold in sterile 0.9% NaCl and cultivated on TSA plates at 37°C for 24 hours. The experiments were performed in triplicate, and the data are presented as mean values with standard deviations.

### Whole-genome sequencing of isolated bacteriophages

The bacteriophage suspensions were treated with DNase I (Norgen Biotek Corp., Canada) to eliminate host DNA. The bacteriophage DNA from OPT-SA02, OPT-SC01, and OPT-SX11 was extracted using a phage DNA isolation kit (Norgen Biotek Corp., Canada). The bacteriophage solutions were prepared at a titer of 10^9^ PFU/mL, and the extraction was carried out according to the manufacturer’s manual. To enhance cell lysis, 4 µL of proteinase K (20 mg/mL, Qiagen) was added to each bacteriophage suspension and incubated at 55°C for 15 minutes. The isolated bacteriophage DNAs were subjected to whole-genome sequencing via the Illumina MiSeq platform (2 × 300 bp paired-end reads) by Sanigen Co., Ltd. (South Korea). Genes were predicted using the Prokaryotic Genome Annotation (Prokka, v.1.14.6) in virus annotation mode (46). BLASTp analysis (90% coverage) was conducted to assign putative functions for each predicted protein on the basis of its sequence homology against the National Center for Biotechnology Information (NCBI) non-redundant protein sequences database (NCBI-nr).

### Genome reconstruction and sequence-based analysis of bacteriophages

The whole genomes of three newly isolated bacteriophages (OPT-SA02, OPT-SC01, and OPT-SX11) were compared with the database of Prokaryotic Virus Remote Homologous Groups (PHROGs) using the UBLAST algorithm implemented in USEARCH software (v.11.0.667), with a threshold of 1e-9 for the E-value and default parameters for the remaining bacteriophages (47–49). This analysis was conducted to categorize coding sequences (CDSs) into functional groups such as lysis-related genes, structural genes, and DNA metabolism genes based on their similarity to entries in the PHROGs database. Rows where the “protein identity” value was equal to or greater than 90% and where both the “query coverage” and “target coverage” values were equal to or greater than 90% were extracted. Among these, the value with the highest bitscore was used. The protein sequences translated from protein-coding genes (CDSs) were used as inputs. Genome statistics were generated via RefSeq GenBank format files (GBFF). The circular visualization of the genomes of the three newly isolated bacteriophages was generated with CIRCOS software (v.0.69-9) (50).

### Pan-genome and phylogenetic analysis

The genomes of 174 bacteriophages, including three newly isolated phages (OPT-SA02, OPT-SC01, and OPT-SX11) and other bacteriophages targeting *Staphylococcus*, were analyzed using Bacterial Pan Genome Analysis (BPGA) v.1.3, which employs the UBLAST function of the USEARCH software (48). This analysis identified orthologous clusters and calculated genetic diversity by categorizing genes into core, accessory, and unique genomes. Protein sequences were compared using a protein identity cutoff of 90%, and the resulting clusters were evaluated to determine shared and unique gene counts among bacteriophages.

The data set included 36 bacteriophages from the family *Herelleviridae*, 22 from the family *Rountreeviridae*, and 113 from an unknown family. Annotations for the remaining 171 bacteriophages, all assembled to the “complete genome” level, were obtained from the public database of NCBI. Phylogenetic analysis was performed using annotated CDSs from the 174 bacteriophage genomes to explore genetic diversity and evolutionary relationships. A neighbor-joining phylogenetic tree was constructed to visualize the evolutionary distances among the bacteriophages.

### Protein structure prediction

The structure of a putative endolysin derived from the newly isolated bacteriophages, confirmed through a genome reconstruction process, was predicted using AlphaFold v.2.3 (41). The locally installed structure prediction pipeline utilized multiple databases, including UniRef90, MGnify, BFD, Uniclust30, PDB70, and PDB (41, 51–56). The prediction process involved five steps: MSA construction, template search, inference with five models, model ranking on the basis of the average confidence metric called the predicted local distance difference test (pLDDT) score, and constrained relaxation of the predicted structures. This reliable pipeline generated five structural models for each target protein sequence, each accompanied by its corresponding pLDDT score, predicted template modeling (pTM) score, and predicted aligned error (PAE) heatmap for model accuracy evaluation. The pLDDT score, a metric that quantifies the distance deviation between the predicted and experimentally determined positions of each amino acid residue in a protein, has been widely used to evaluate the precision of protein structure prediction (41, 57). For the predicted structure of the endolysin derived from newly isolated bacteriophages, the pLDDT score was interpreted as follows (41): scores > 90 indicated high accuracy; 70–90 generally indicated good accuracy; 50–70 implied low confidence; and <50 was indicative of disordered or unstructured regions.

The query structure for the putative endolysin derived from three newly isolated bacteriophages was searched using the Foldseek web server (58). The predicted protein structure was further compared and aligned with the experimentally validated protein structure on the basis of the Foldseek search result.

### Statistical analysis

The statistical significance of bacteriophage stability, antibacterial activity, and potential efficacy in milk was evaluated using one-way analysis of variance (ANOVA) and Tukey’s honestly significant difference (HSD) test. All experiments were conducted in triplicate, and differences among conditions were assessed using Tukey’s HSD test. All data were analyzed using the SciPy and Statsmodels libraries, and statistical significance was denoted as follows: *P* < 0.05 (*), *P* < 0.01 (**), and *P* < 0.001 (***). For thermal stability, statistical analyses were conducted to compare the bacteriophage titers at 40°C, 50°C, 60°C, and 70°C against the reference temperature of 35°C at each time point (0, 10, 30, and 60 min). For the statistical analysis of pH stability, the titers under neutral conditions (pH 7) were used as a reference for comparison with those under acidic (pH 3 and pH 5) and alkaline conditions (pH 9 and pH 11). For antibacterial activity, the effect at an MOI of 0.01 was compared with that of the untreated control (control). The MOI of 0.01 was selected as the lowest tested bacteriophage concentration, enabling the indirect inference of the effectiveness of higher concentrations (MOI 0.1 and MOI 1). For the evaluation of bacteriophage efficacy in milk, the bacterial growth inhibitory effects were compared between the control group and the bacteriophage-treated groups at each time point (0, 2, and 6 h).

## RESULTS

### Physiological characterization of newly isolated bacteriophages

**Fig 1:**
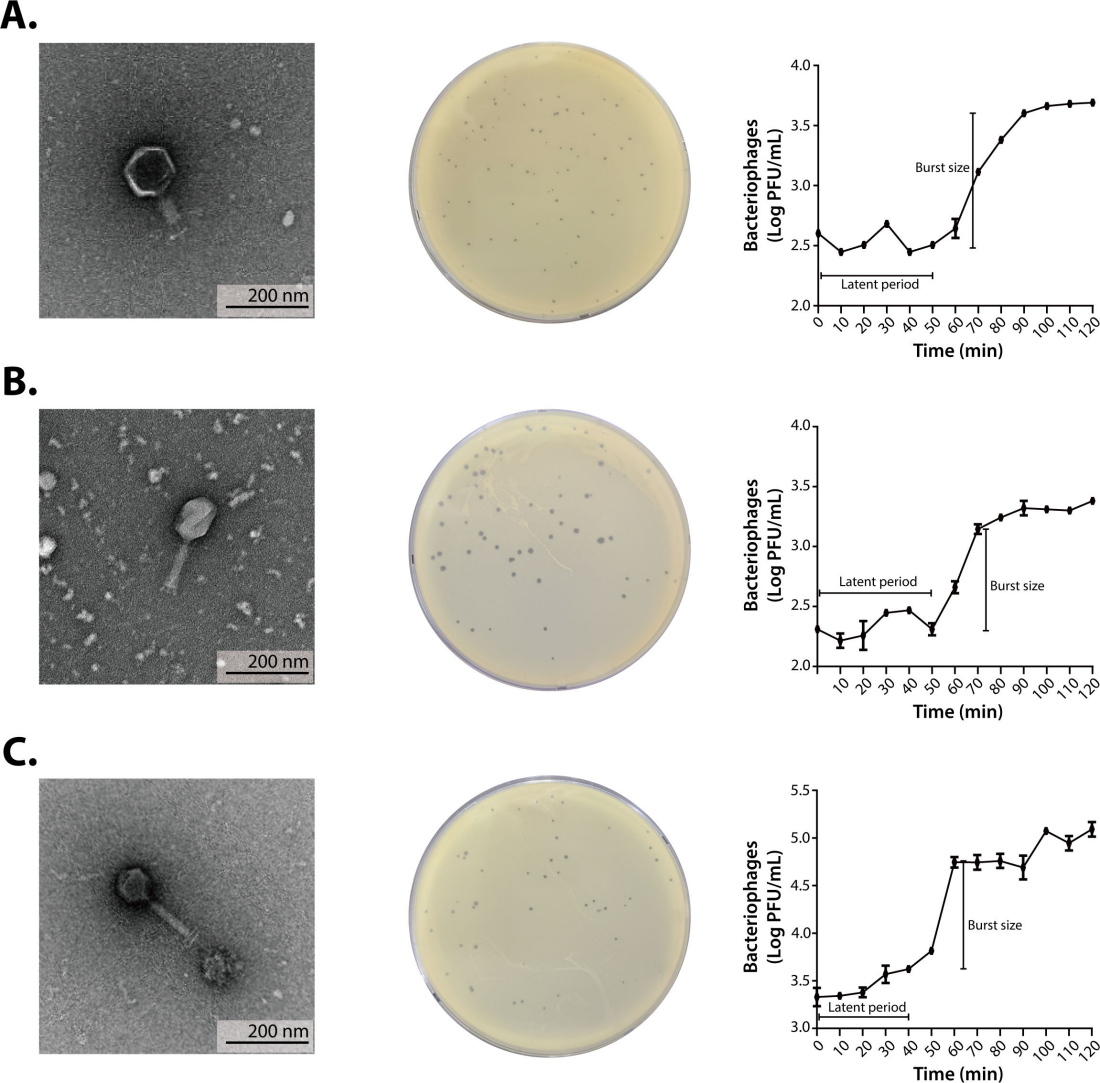
Characterization of isolated bacteriophages OPT-SA02, OPT-SC01, and OPT-SX11 infecting *Staphylococcus* species. Each section describes one of the isolated bacteriophages: OPT-SA02, OPT-SC01, and OPT-SX11, respectively. (**A**) The transmission electron microscope images showing the morphologies of the isolated bacteriophages. The scale bar represents 200 nm. (**B**) Plaque assays illustrating the lysis patterns and plaque formation. (**C**) One-step growth curves indicating the latent periods and burst sizes of the three newly isolated bacteriophages OPT-SA02, OPT-SC01, and OPT-SX11. The latent periods for these bacteriophages are approximately 50, 50, and 40 min, respectively. The burst sizes are estimated to be 14 PFU per infected cell for OPT-SA02, 10 PFU per infected cell for OPT-SC01, and 28 PFU per infected cell for OPT-SX11. Error bars represent standard deviations from three independent experiments.

Three bacteriophages, designated OPT-SA02, OPT-SC01, and OPT-SX11, were isolated from chicken fecal and sewage samples in Chungcheongbuk-do, South Korea, using *S. aureus* or *S. xylosus* as indicator strains. TEM analysis revealed that all three bacteriophages had icosahedral heads and long tails, with head dimensions of 108 ± 5, 110 ± 5, and 104 ± 5 nm, and tail lengths of 133 ± 5, 141 ± 5, and 204 ± 5 nm for OPT-SA02, OPT-SC01, and OPT-SX11, respectively (Fig. 1). These morphological characteristics suggest that bacteriophages exhibit the morphology of myoviruses, which are known to infect a wide range of bacterial hosts, including staphylococci.

One-step growth curves were constructed to determine the burst size and latent period of the three newly isolated bacteriophages using *S. aureus* or *S. xylosus*. The burst size, which indicates the number of new virions produced per infected cell, and the latent period, representing the time taken from bacteriophage adsorption to the first release of viral progeny, are critical parameters in evaluating bacteriophage efficacy. Larger burst sizes and short latent periods typically reflect potent lytic activity, making these bacteriophages promising candidates for antibacterial applications (59). The burst sizes were 14, 10, and 28 PFU per infected cell for OPT-SA02, OPT-SC01 and OPT-SX11, respectively, with latent periods of 40 minutes for OPT-SA02 and 50 minutes for both OPT-SC01 and OPT-SX11 (Fig. 1).

To effectively utilize bacteriophages as therapeutic agents, it is crucial to assess their stability under various environmental conditions (Fig. S1). The stability of the isolated bacteriophages was evaluated across a wide range of pH (2–12) and temperature (37–70°C) conditions. OPT-SA02 and OPT-SC01 maintained stability at pH 5–10 and temperatures ranging from 35°C to 50°C. At 60°C, a reduction of approximately 10^6^ and 10^2^ PFU/mL in phage titer was observed after 1 h of treatment for OPT-SA02 and OPT-SC01, respectively. OPT-SA02 showed a reduction of approximately 10^5^ PFU/mL at pH 4 than 10^1^ PFU/mL at pH 11. OPT-SX11 demonstrated a broader pH stability (pH 4–11) than the other bacteriophages did, while its temperature stability was similar (35–50°C). At 60°C, OPT-SX11 resulted in a reduction in the phage titer of 10^3^ PFU/mL. The stability of these bacteriophages under various pH and temperature conditions suggests their potential for application in diverse settings, including the treatment of bovine mastitis.

The host range specificity of the newly isolated bacteriophages was determined by spot testing their infectivity against multiple gram-positive bacteria (Table 1; Tables S1 and S2). The results revealed that the bacteriophages specifically infected *Staphylococcus* species. To further confirm host range specificity, additional spot tests were conducted on six non-*Staphylococcus* gram-positive bacteria and four gram-negative bacteria, which are commonly associated with food safety and dairy farm environments (Table 2) (60, 61). These included pathogens such as *Bacillus cereus* and *Escherichia coli*, which have significant implications in foodborne illnesses and animal infections. The results indicated no lytic activity against any of the non-*Staphylococcus* strains tested.

To assess the efficacy of the isolated bacteriophages in controlling their respective host strains, the antibacterial activity of each bacteriophage was evaluated against *S. aureus* (OPT-SA02) or *S. xylosus* (OPT-SC01 and OPT-SX11) at different MOI levels (1.0, 0.1, and 0.01) after 3 hours of incubation (Fig. S1). The OD600 of the phage-treated groups was significantly lower than the initial OD600, indicating that the growth of *Staphylococcus* species was inhibited by these bacteriophages regardless of the MOI levels. In contrast, the negative control (bacterial culture without bacteriophages) continued to grow during incubation, reaching bacterial cell densities of approximately 5.0 and 3.5 for *S. aureus* and *S. xylosus*, respectively (62). These results demonstrate the strong antibacterial activity of these newly isolated bacteriophages against their respective host strains, even at low MOI levels.

To evaluate the potential of the isolated bacteriophages as a treatment for bovine mastitis, each bacteriophage was individually added to ultra-high temperature-treated whole milk containing 10^5^ CFUs of *S. aureus* (OPT-SA02) or *S. xylosus* (OPT-SC01 and OPT-SX11) (Fig. S2). The number of viable cells was monitored over a 6-hour period at an MOI of 1.0. After 6 hours, bacterial counts were significantly reduced in the bacteriophage-treated groups compared to the control group: OPT-SA02 reduced *S. aureus* counts by approximately 52.22%, OPT-SC01 reduced *S. xylosus* counts by 49.40%, and OPT-SX11 achieved a 44.05% reduction in *S. xylosus* counts.

### Genomic characterization of newly isolated bacteriophages

The genetic characteristics of these three newly isolated bacteriophages, OPT-SA02, OPT-SC01, and OPT-SX11, were annotated with the Prokaryotic Genome Annotation (Prokka) pipeline (46), and BLASTp analysis against the NCBI-nr database was performed to assign putative functions to each predicted open reading frame (ORF) (63) (Table S3). These bacteriophages were identified as linear contigs with genome sizes of 141,852, 142,961, and 140,468 bp, respectively. They presented coding densities of 89.06%, 84.79%, and 89.55% and G+C contents of 30.15%, 30.93%, and 30.17%, respectively. Genome characterization revealed that OPT-SA02, OPT-SC01, and OPT-SX11 contained 228, 211, and 226 ORFs, and possessed 4, 1, and 4 tRNAs, respectively.

**Fig 2:**
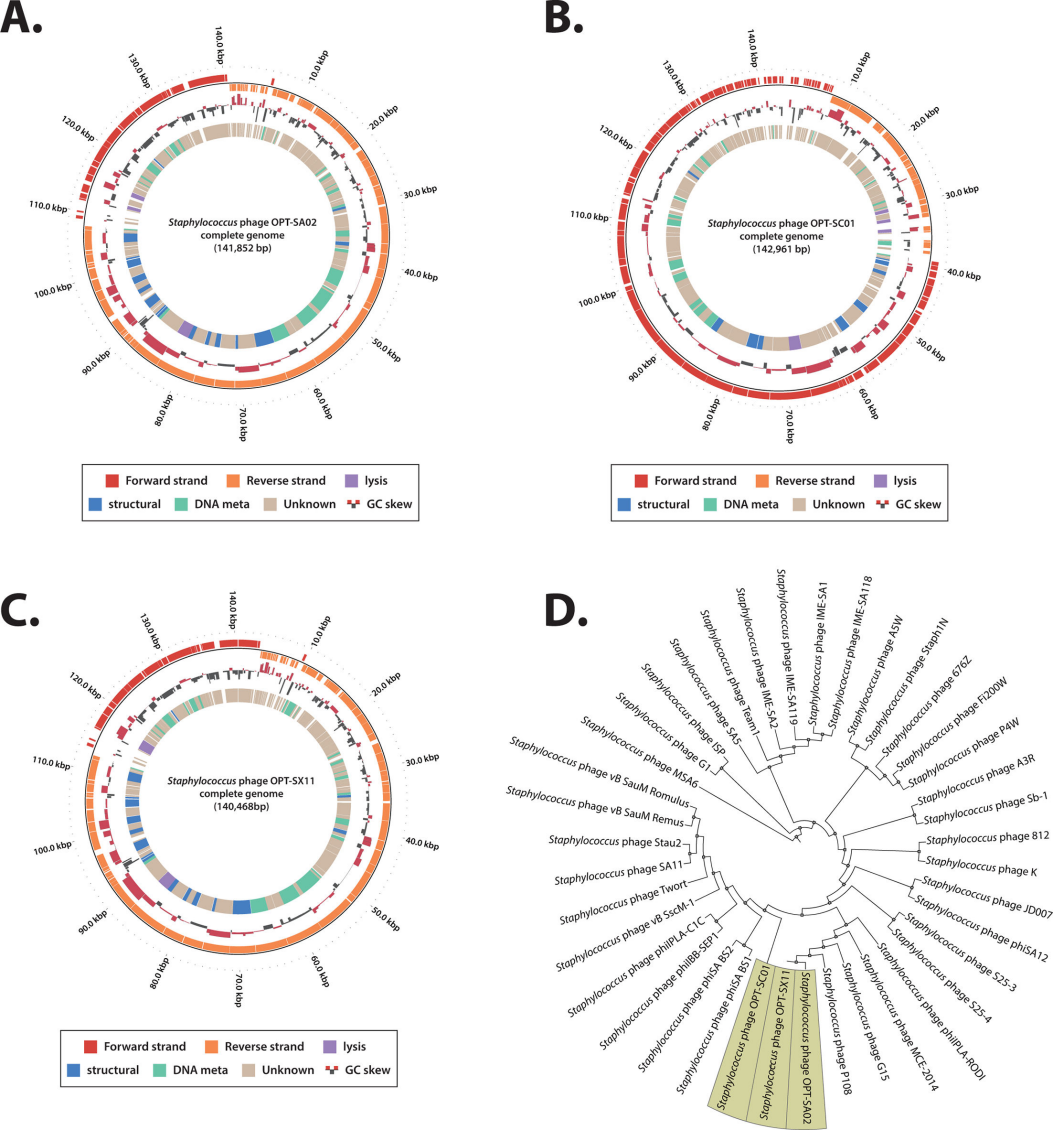
Genomic characterization of isolated bacteriophages. The circular representation of the three newly isolated bacteriophages OPT-SA02 (**A**), OPT-SC01 (**B**), and OPT-SX11 (**C**). The outmost circles indicate the organization of contigs for genome annotation, with circles for forward (red) and reverse (orange) strands. The next inner circles indicate the GC skew of each genome. The innermost circles indicate multiple genomic features color coded as follows: lysis-related genes (purple), structural genes (blue), DNA metabolism genes (green), and genes of unknown function (gray). (**D**) A phylogenetic tree showing the relationships between the three newly isolated phages (highlighted in yellow) and 36 *Staphylococcus* phages belonging to the *Herelleviridae* family. The whole genome sequences were used to construct the phylogenetic tree, illustrating evolutionary distances and clustering patterns among the bacteriophages.

The genomic maps of the three isolated bacteriophages derived from Prokka and BLASTp were classified into four functional groups on the basis of local alignment with the PHROG database (Tables S4 to S6): DNA and RNA metabolism genes, host-lysis genes, structural genes, and functionally unknown genes (Fig. 2A through C). Lysis-related genes were identified in all three bacteriophages: holin (OPT_SA02_00830 in OPT-SA02, OPT_SC01_00335 in OPT-SC01, and OPT_SX11_00895 in OPT-SX11) and endolysin (OPT_SA02_00845 in OPT-SA02, OPT_SC01_00325 in OPT-SC01, and OPT_SX11_00900 in OPT-SX11). The protein-coding genes, OPT_SA02_00830 and OPT_SA02_00845 in OPT-SA02, OPT_SC01_00325 and OPT_SC01_00335 in OPT-SC01, and OPT_SX11_00895 and OPT_SX11_00900 in OPT-SX11, were classified as lytic cassettes responsible for hydrolyzing their host bacterial cell wall. This classification was based on the typical arrangement of bacteriophage lytic cassettes in a continuous sequence within a cluster.

To further understand the evolutionary relationships and genetic diversity of the three newly isolated bacteriophages, comprehensive phylogenetic and pan-genome analyses were performed. The phylogenetic analysis included the genomes of 174 bacteriophages, encompassing the three newly isolated bacteriophages. All the genomes were annotated as “complete” in the RefSeq database, with *Staphylococcus* specifically targeted as their host (Fig. S3). Through this analysis, 36 bacteriophages were identified as belonging to the *Herelleviridae*, 22 to the *Rountreeviridae*, and 113 to an unknown family. The pan-genome analysis revealed the absence of a core genome, with 13,507 genes forming 1,781 clustering groups constituting the accessory genome (86.54%) and 2,100 unique genes (13.46%).

Based on the phylogenetic analysis of bacteriophages infecting *Staphylococcus* species, the genetic composition of 29 bacteriophages, including the three newly isolated bacteriophages, within the same clade was investigated (Fig. S3B; Table S7). The analysis revealed that 1,914 genes (32.05%) formed 66 clustering groups shared among all 29 bacteriophages. Additionally, 3,833 genes (64.19%) constituting 305 clustering groups were found in some but not all bacteriophages, representing diverse genetic contents, whereas 224 genes (3.75%) were unique to individual bacteriophages. Interestingly, a group of genes encoding lysin (OPT_SA02_00845 in OPT-SA02, OPT_SC01_00325 in OPT-SC01, and OPT_SX11_00900 in OPT-SX11) was identified as a conserved genetic backbone shared by all bacteriophages within the clade. Furthermore, a lytic cassette, holin, was detected in the group closely related to the three newly isolated bacteriophages, present in all three but shared by 96.55% (28/29) of the bacteriophages within the group. Upon examining the genetic characteristics of the three newly isolated bacteriophages within this group, OPT-SC01 was found to have a relatively distinct genetic composition, with 99 out of its 211 protein-coding genes being unique. In contrast, OPT-SX11 shared more common genetic features, with 66 out of its 226 genes shared by all other bacteriophages in this group. To further understand the genetic diversity and relationships among bacteriophages within the families closely related to the three newly isolated bacteriophages, a detailed pan-genome analysis was performed on bacteriophages belonging to the *Herelleviridae* and *Rountreeviridae* (Fig. S3C). The results revealed that no gene groups were commonly shared by all bacteriophages in either family, suggesting that the genetic composition of bacteriophages is highly diverse, even within the same family.

### Protein structure analysis for putative endolysins

A BLASTp search with the NCBI and PHROG databases identified OPT_SA02_00845, OPT_SC01_00325, and OPT_SX11_00900 from the newly isolated bacteriophages OPT-SA02, OPT-SC01, and OPT-SX11, respectively, as putative endolysin sequences. These are phage-derived lytic proteins, suggesting that the three newly isolated bacteriophages possess genes encoding endolysins, which are essential for their lytic activity against bacterial host cells. This finding indicates their potential function as lytic bacteriophages.

**Fig 3:**
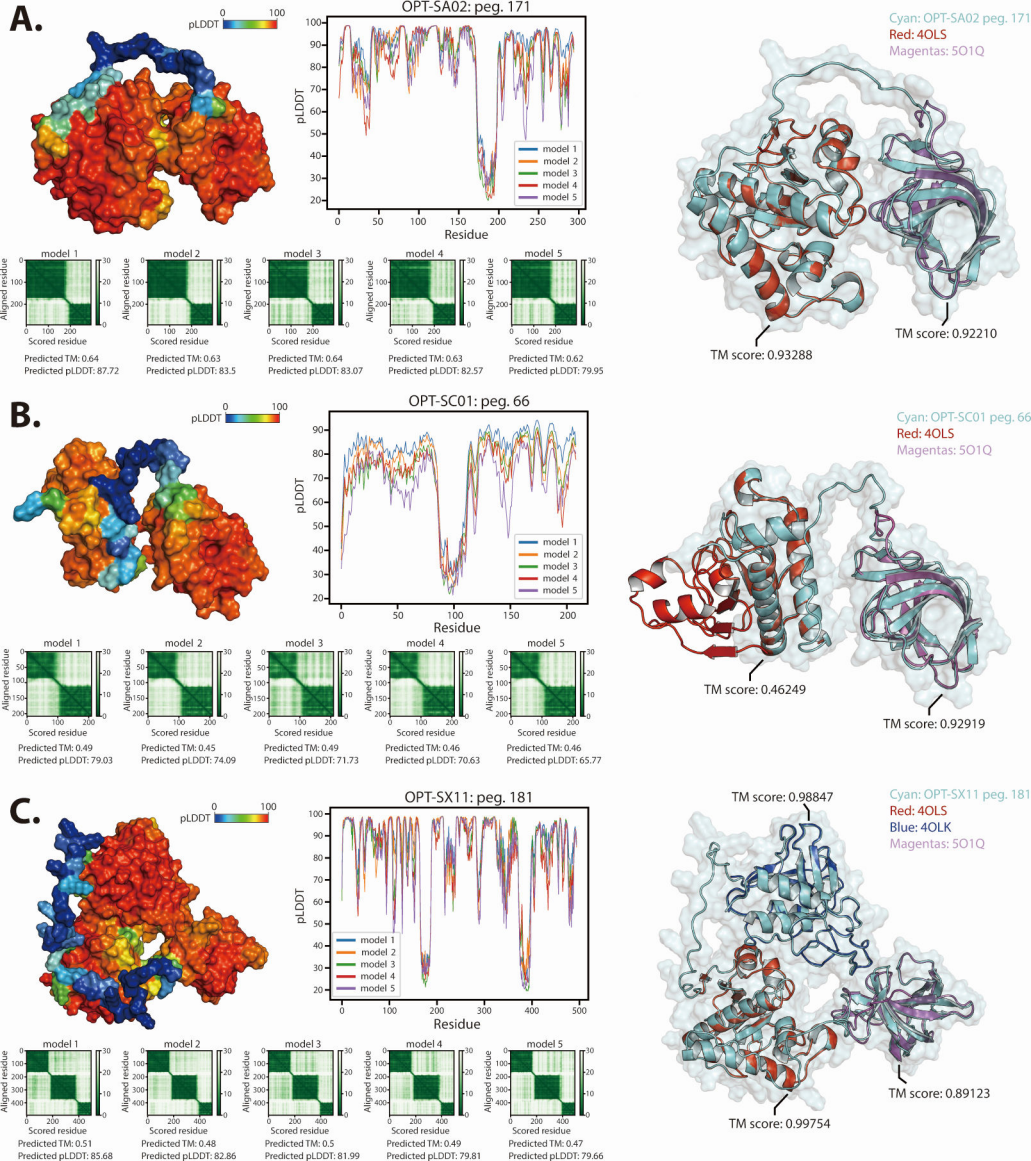
Characterization of putative endolysin from the newly isolated bacteriophages based on structural prediction. The results of 3D modeling and comparative analysis of putative endolysin from three newly isolated bacteriophages, OPT-SA02 (**A**), OPT-SC01 (**B**), and OPT-SX11 (**C**), respectively. Each section details the endolysins discovered in isolated phages, with 3D structural predictions compared to reference structures identified using Foldseek. Structural predictions were performed using AlphaFold2.3, and structural alignments were conducted via the TM-align server. The 3D surface structures of the predicted models are color coded according to pLDDT score, and 2D plots show the pLDDT scores for each residue. The PAE maps of the five predicted models indicate the predicted error between all residue pairs. A lower value (dark green) indicates higher prediction reliability. The results of structural alignment of the predicted endolysin with reference structures retrieved from Foldseek. The reference structures are color coded, and the TM scores indicate the quality of the alignment.

To further characterize the putative endolysins, a highly reliable protein structure prediction pipeline was utilized to generate predicted structures of these proteins (Table S8) (41, 42). The results showed that the average pLDDT scores of the five predicted models for each putative endolysin from OPT-SA02, OPT-SC01, and OPT-SX11 were 80.96, 69.69, and 78.56, respectively (Fig. 3). These scores indicate moderate levels of confidence in the predicted structures. Notably, the predicted protein structures of the endolysins from the three bacteriophages displayed multi-domain architectures with multiple functional domains connected by linker sequences.

To determine reference structures for the protein domains and elucidate the structural features of the endolysins derived from the isolated bacteriophages, the structural alignment tool Foldseek was used (58). This analysis confirmed that the endolysins of OPT-SA02 and OPT-SC01 consist of the LysGH15 amidase domain (PDB ID: 4OLS) and the LysF1 SH3b domain (PDB ID: 5O1Q) (64, 65). Furthermore, the endolysin of OPT-SX11 was found to include the LysGH15 CHAP domain (PDB ID: 4OLK), the LysGH15 amidase domain, and the LysF1 SH3b domain (64). Analysis of the 4OLS structure, which is common to the endolysins derived from the three bacteriophages, identified the domain as an N-acetylmuramoyl-L-alanine amidase, sharing the same structure as the λ prophage Ba02 endolysin PlyL (66). These findings suggest that each endolysin possesses catalytic activity to cleave the amide bond between *N*-acetylglucosamine and the L-alanine moiety on the stem peptide in peptidoglycan. Structural alignment analysis revealed that the N-acetylmuramoyl-L-alanine amidase domain of the OPT-SC01 endolysin did not completely align with the reference structure (PDB ID: 4OLS), resulting in a relatively low template modeling (TM) score (0.46). However, detailed analysis of the active site residues demonstrated that the catalytic core of the OPT-SC01 endolysin was preserved (Fig. S4). Considering the bacteriophage susceptibility test results and the established role of amidase domains in *Staphylococcus* endolysins, which are primarily associated with cell wall binding rather than direct catalytic activity, these findings suggest that the OPT-SC01 endolysin maintains lytic activity, despite partial structural misalignment (67).

Interestingly, it was confirmed that all three bacteriophage-derived endolysins possess the LysF1 SH3b domain, a cell wall-binding domain (CBD). This domain has been shown to mediate peptidoglycan binding by recognizing ligand molecules within the bacterial cell wall, contributing to substrate specificity in staphylococcal endolysins (17). Additionally, it directly interacts with peptidoglycan components, further enhancing substrate specificity (68).

## DISCUSSION

The persistent threat posed by multidrug-resistant bacteria, particularly *Staphylococcus* species, remains a significant global health challenge, emphasizing the critical need for novel therapeutic strategies and tools to combat these pathogens. In this study, three newly isolated lytic bacteriophages (OPT-SA02, OPT-SC01, and OPT-SX11) were characterized using multiple analytical approaches. These bacteriophages were specific to *Staphylococcus* species and demonstrated stability across a wide range of pH and temperature conditions, suggesting their potential for application in diverse physiological environments. The susceptibility of these three bacteriophages indicates activity against staphylococci, including *Staphylococcus aureus* (both methicillin-susceptible and methicillin-resistant strains) and coagulase-negative *Staphylococcus* sp. such as *Staphylococcus epidermidis*, *Staphylococcus chromogenes*, and other strains. Hence, three newly isolated bacteriophages present a broad spectrum of strains within the target species.

The bacteriophages demonstrated specificity toward *Staphylococcus* species, as shown by spot tests revealing that these phages did not lyse other gram-positive or gram-negative bacteria. This narrow host range is advantageous for phage therapy as it minimizes potential impacts on beneficial bacteria within the host microbiome. By selectively targeting *Staphylococcus* species, these bacteriophages are less likely to disrupt other commensal and beneficial microorganisms, thereby preserving the natural microbial balance in dairy environments. Notably, antibacterial activity was observed across a range of multiplicities of infection, and their ability to effectively reduce bacterial counts in milk environments indicates potential applications in treating bovine mastitis. This capability could lead to improvements in animal health, milk quality, and reductions in economic losses associated with mastitis.

Genomic analysis provided crucial insights into the potential applications and evolutionary relationships of the bacteriophages OPT-SA02, OPT-SC01, and OPT-SX11. Despite being isolated from different environments, these bacteriophages displayed conserved genome structures and shared characteristics with other *Staphylococcus*-infecting phages, suggesting that their genetic composition is suitable for treating *Staphylococcus* infections. Notably, lysis-related genes, such as holin and endolysin, were identified in all three bacteriophages and were found to form lytic cassettes. These findings support the ability of these bacteriophages to effectively lyse host bacteria, further enhancing their potential as therapeutic agents.

Phylogenetic and pan-genome analyses offered valuable insights into the genetic diversity and evolutionary relationships of these bacteriophages. Phylogenetic analysis of *Staphylococcus*-infecting phages revealed that OPT-SA02, OPT-SC01, and OPT-SX11 are closely related to the *Herelleviridae* family. Interestingly, while TEM analysis indicated myovirus structures, phylogenetic analysis suggested that these bacteriophages are more closely related to *Herelleviridae* in terms of genome composition, gene organization, and evolutionary relationships (69–71). The absence of a core genome among these bacteriophages suggests notable genetic diversity, which is consistent with known characteristics of the *Herelleviridae* family. This genetic diversity may be related to the acquisition of various genetic elements through horizontal gene transfer, potentially contributing to their adaptability and ecological success (72, 73).

The structural characteristics of the endolysins discovered in the newly isolated bacteriophages OPT-SA02, OPT-SC01, and OPT-SX11 provide insights into their multifunctionality and potential applications. The multi-domain structures observed in these endolysins, particularly the combination of LysGH15 amidase (N-acetylmuramoyl-L-alanine) and SH3b domains, suggest their multifunctionality. The additional LysGH15 CHAP domain found in the OPT-SX11 endolysin further expands the functional diversity of this protein. Interestingly, while the OPT-SC01-derived endolysin had relatively low pLDDT scores and incomplete alignment with reference structures, further analysis confirmed that its active site was preserved.

The discovery of identical CBDs in all three endolysins is particularly significant, as CBDs confer substrate specificity by recognizing and binding to specific ligand molecules in the bacterial cell wall. This shared presence of CBD suggests that the endolysins derived from the isolated bacteriophages may specifically target the same *Staphylococcus* species, a finding supported by host range test results demonstrating this specificity.

Although this study demonstrates the potential of bacteriophages as antibacterial agents against *Staphylococcus aureus* and *Staphylococcus xylosus* in controlled environments, applications in raw milk environments present additional challenges. The use of UHT-treated milk in this study allowed for controlled evaluation of bacteriophage efficacy by minimizing interference from microbial contaminants. This approach provides a standardized baseline for assessing antibacterial activity. However, the complex microbiota and biochemical environment of raw milk may affect the activity, stability, and host specificity of the bacteriophages. Future studies are needed to evaluate the efficacy of these bacteriophages in raw milk, which may require optimizing phage formulations or developing phage cocktails to account for the diversity of potential bacterial strains and environmental factors. These findings highlight the importance of assessing phage-based therapeutics under real-world conditions to better understand their potential in dairy applications.

### Conclusion

This study provides a detailed characterization of the three newly isolated bacteriophages, OPT-SA02, OPT-SC01, and OPT-SX11, specifically targeting *Staphylococcus* species associated with bovine mastitis. Combined genomic and morphological analyses confirmed these phages belong to the *Herelleviridae* family and possess stable lytic activity and narrow host specificity, making them promising candidates for phage therapy. The consistent presence of cell wall-binding domains among these phages suggests their targeted efficacy against *Staphylococcus* species, with minimal impact on beneficial microbiome within dairy environments. These findings highlight the potential of bacteriophages as alternative treatments for antibiotic-resistant *Staphylococcus* infections while also underscoring the need for further research to optimize phage formulations for use in complex dairy environments.

## Data Availability

The complete genome sequences and annotations of the three newly isolated bacteriophages have been deposited in GenBank under the accession numbers PQ046923, PQ046924, and PQ046925, respectively.

## SUPPLEMENTAL MATERIAL

Supplemental figures jvi.01901-24-s0001.docx Fig. S1 to S4 and all supplemental legends.

Supplemental tables jvi.01901-24-s0002.xlsx Tables S1 to S8.

ASM does not own the copyrights to Supplemental Material that may be linked to, or accessed through, an article. The authors have granted ASM a non-exclusive, world-wide license to publish the Supplemental Material files. Please contact the corresponding author directly for reuse.
