# Supplementary material for: Characterization and therapeutic potential of newly isolated bacteriophages against Staphylococcus species in bovine mastitis
Source: J Virol. 2025 Feb 14;99(3):e01901-24. doi: 10.1128/jvi.01901-24 (PMC11915829; doi:10.1128/jvi.01901-24)
Supplement: Supplemental figures — Fig. S1 to S4 and all supplemental legends. [file jvi.01901-24-s0001.docx]

**Supplementary information**

**Characterization and therapeutic potential of newly isolated bacteriophages against *Staphylococcus* species in bovine mastitis**

Jae-hyun Cho^a, #^, Gyu Min Lee^b, #^, Seyoung Ko^b, *^, Youngju Kim^a, *^, Donghyuk Kim^b, *^

^a^Optipharm Inc., Cheongju-si, Chungcheongbuk-do, Republic of Korea

^b^School of Energy and Chemical Engineering, Ulsan National Institute of Science and Technology (UNIST), Ulsan 44919, Republic of Korea

^#^ These authors have contributed equally to this work and share first authorship.

*Corresponding authors:

E-mail address: dkim@unist.ac.kr (Donghyuk Kim), kimyj@optipharm.co.kr (Youngju Kim), sierrayk@unist.ac.kr (Seyoung Ko)

Tel: +82-52-217-2945 (Donghyuk Kim), +82-42-249-7542 (Youngju Kim), +82-52-217-3553 (Seyoung Ko)

Fax: +82-52-217-3009 (Donghyuk Kim, Seyoung Ko), +82-42-249-7501 (Youngju Kim)

**Supplementary data**

**Figure S1. Temperature and pH stability profiles, and bactericidal effects of bacteriophages OPT-SA02, OPT-SC01, and OPT-SX11.**

**Figure S2. Viable cell counts of *S. aureus* and *S. xylosus* in milk treated with bacteriophages.**

**Figure S3. Pan-genome analysis of *Staphylococcus*-infecting phages at different taxonomic levels.**

**Figure S4. Structural analysis of the predicted N-acetylmuramoyl-L-alanine amidase domain from OPT-SC01 phage endolysin.**

**Table S1. Sources and characteristics of *Staphylococcus aureus* strains used in host specificity testing.**

**Table S2. Sources and characteristics of Coagulase-Negative *Staphylococci* (CoNS) strains used in host specificity testing.**

**Table S3. Genomic features of newly isolated bacteriophages.**

**Table S4. The functional annotation of isolated bacteriophage OPT-SA02.**

**Table S5. The functional annotation of isolated bacteriophage OPT-SC01.**

**Table S6. The functional annotation of isolated bacteriophage OPT-SX11.**

**Table S7. Pan-genome classification of *Staphylococcus*-infecting phages.**

**Table S8. The results of domain investigation of the three newly isolated bacteriophage-derived endolysins.**

**
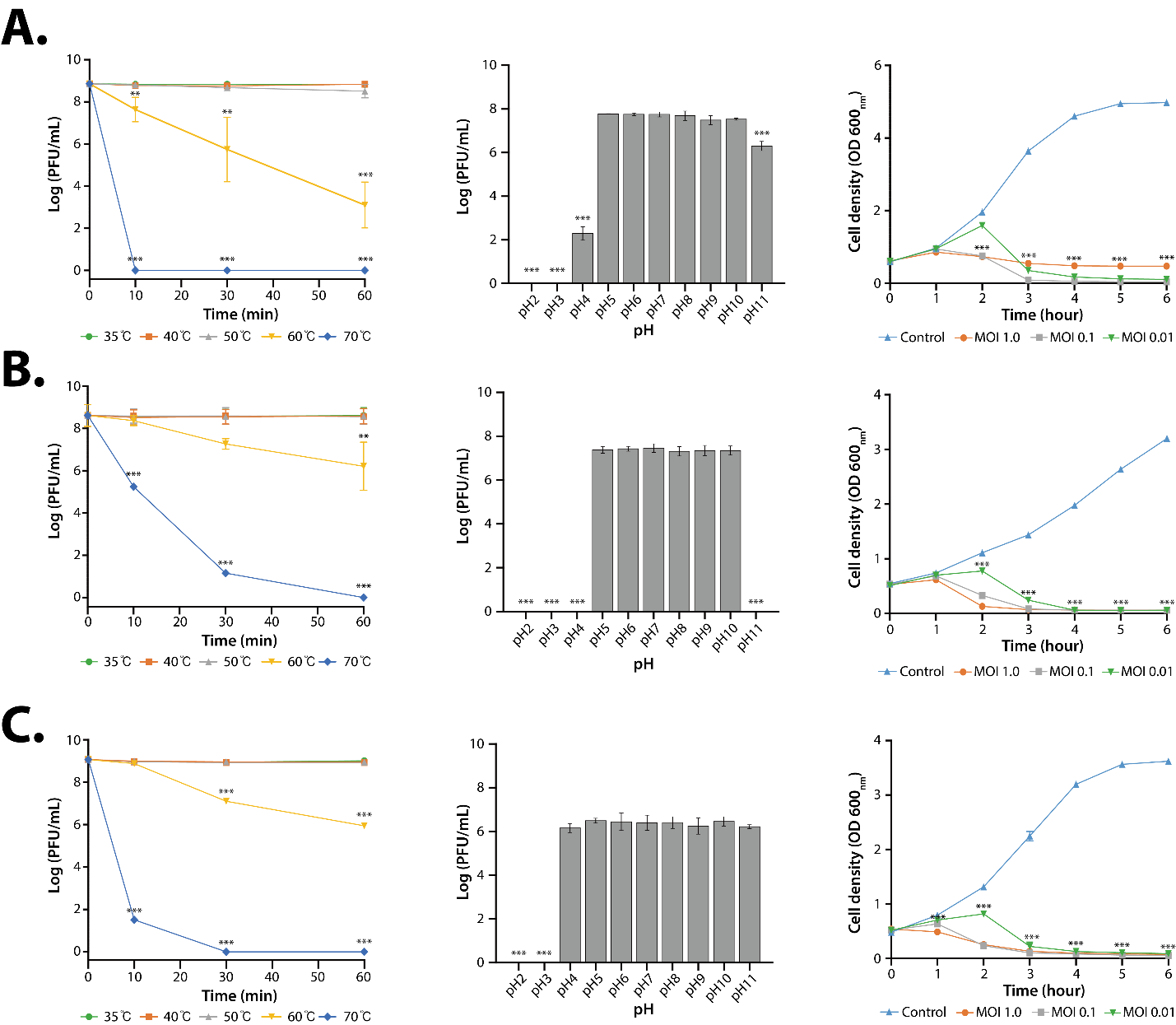
**

**Figure S1. Temperature and pH stability profiles, and bactericidal effects of bacteriophages OPT-SA02, OPT-SC01, and OPT-SX11.**

(A) OPT-SA02 bacteriophage stability and growth characteristics. The left panel shows thermal stability at various temperatures (35°C, 40°C, 50°C, 60°C, and 70°C) over time. The middle panel displays pH stability across different pH values. The right panel illustrates the bactericidal effect through a one-step growth curve at different multiplicities of infection (MOIs). (B) OPT-SC01 bacteriophage stability and growth characteristics. The left panel demonstrates thermal stability, the middle panel shows pH stability, and the right panel presents the bactericidal effect through a one-step growth curve. (C) OPT-SX11 bacteriophage stability and growth characteristics. The panels show thermal stability, pH stability, and bactericidal effect, respectively, arranged in the same order as the previous two bacteriophages. In all thermal stability and pH stability graphs, the y-axis represents Log PFU/mL. For the one-step growth curves, the y-axis shows cell density (OD600). The control and different MOIs (1.0, 0.1, 0.01) in the growth curves are represented by distinct colors. Statistical significance was determined using one-way ANOVA followed by Tukey’s HSD test. Significant differences are indicated as p < 0.05 (*), p < 0.01 (**), and p < 0.001 (***).

**
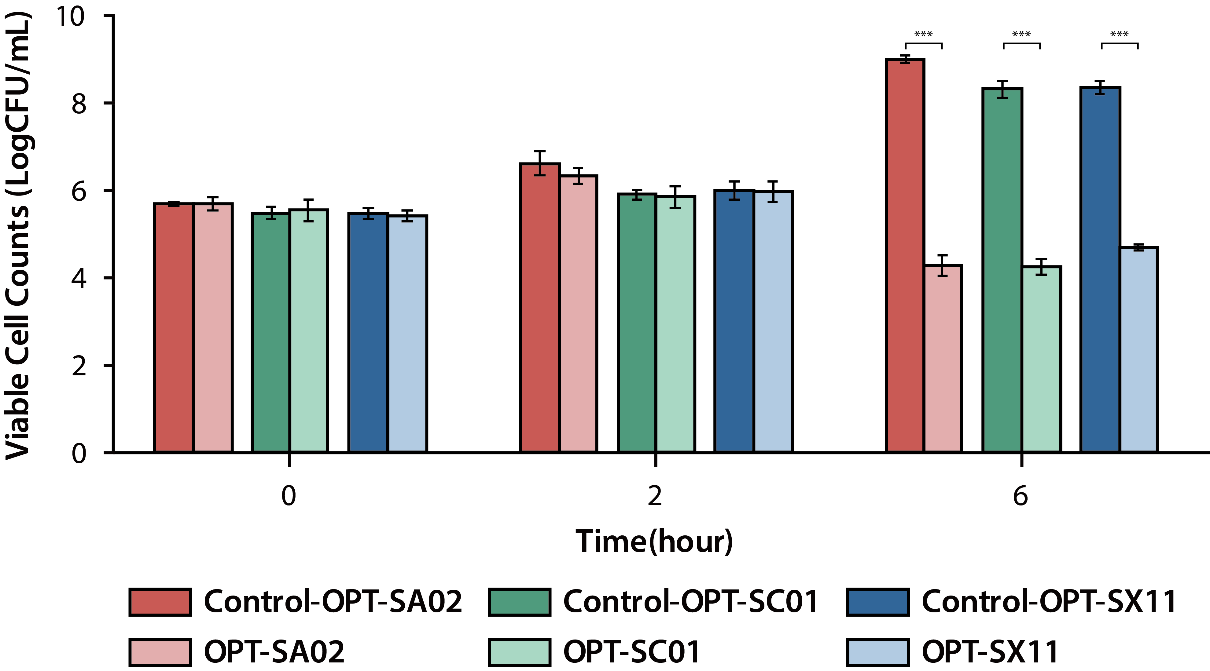
**

**Figure S2. Viable cell counts of *S. aureus* and *S. xylosus* in milk treated with bacteriophages.**

Viable cell counts (Log CFU/mL) of *S. aureus* (OPT-SA02) and *S. xylosus* (OPT-SC01 and OPT-SX11) were monitored over a 6-hour period in ultra-high temperature (UHT)-treated whole milk, both in untreated control groups and groups treated with bacteriophages. The control groups, which included only bacterial cultures without bacteriophages (untreated), are represented by red, green, and blue bars for OPT-SA02, OPT-SC01, and OPT-SX11, respectively. The treated groups (OPT-SA02, OPT-SC01, and OPT-SX11) are shown in pink, light green, and light blue bars, respectively. The error bars indicate standard deviations from three independent experiments. Statistical significance was determined using one-way ANOVA followed by Tukey’s HSD test. Significant differences are indicated as p < 0.05 (*), p < 0.01 (**), and p < 0.001 (***).

**
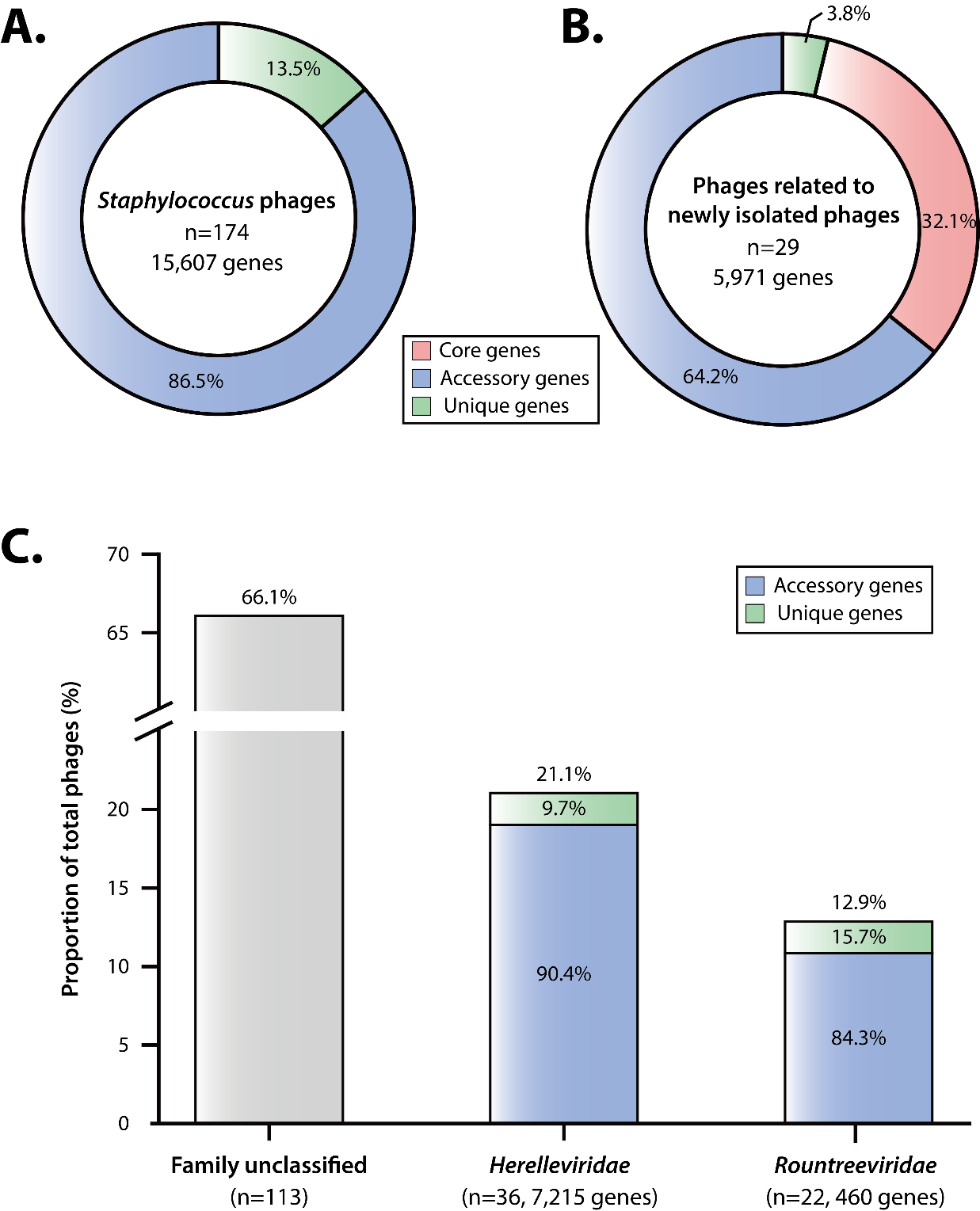
**

**Figure S3. Pan-genome analysis of *Staphylococcus*-infecting phages at different taxonomic levels.**

1. Pan-genome composition of all 174 analyzed *Staphylococcus*-infecting phages, comprising 15,607 genes. The pie chart shows the distribution of core genes (red), accessory genes (blue), and unique genes (green). (B) Pan-genome composition of 29 phages phylogenetically related to the newly isolated phages, totaling 5,971 genes. The pie chart displays the proportion of core genes (red), accessory genes (blue), and unique genes (green) within this subset. (C) Taxonomic distribution of bacteriophages and their pan-genome composition across families. The height of each bar represents the proportion of strains in each family relative to the total number of strains, with *Herelleviridae*, *Rountreeviridae*, and unclassified phages. The colors within each bar are identical to those in panels A and B, representing accessory genes (blue), and unique genes (green). Percentages within each bar represent the relative proportions of these gene categories within the family. The number of phages (n) and total gene count are provided for each family analyzed. For unclassified bacteriophages, pan-genome analysis was not performed due to a lack of sufficient classification criteria, and only the number of phages is shown, represented by a grey bar.


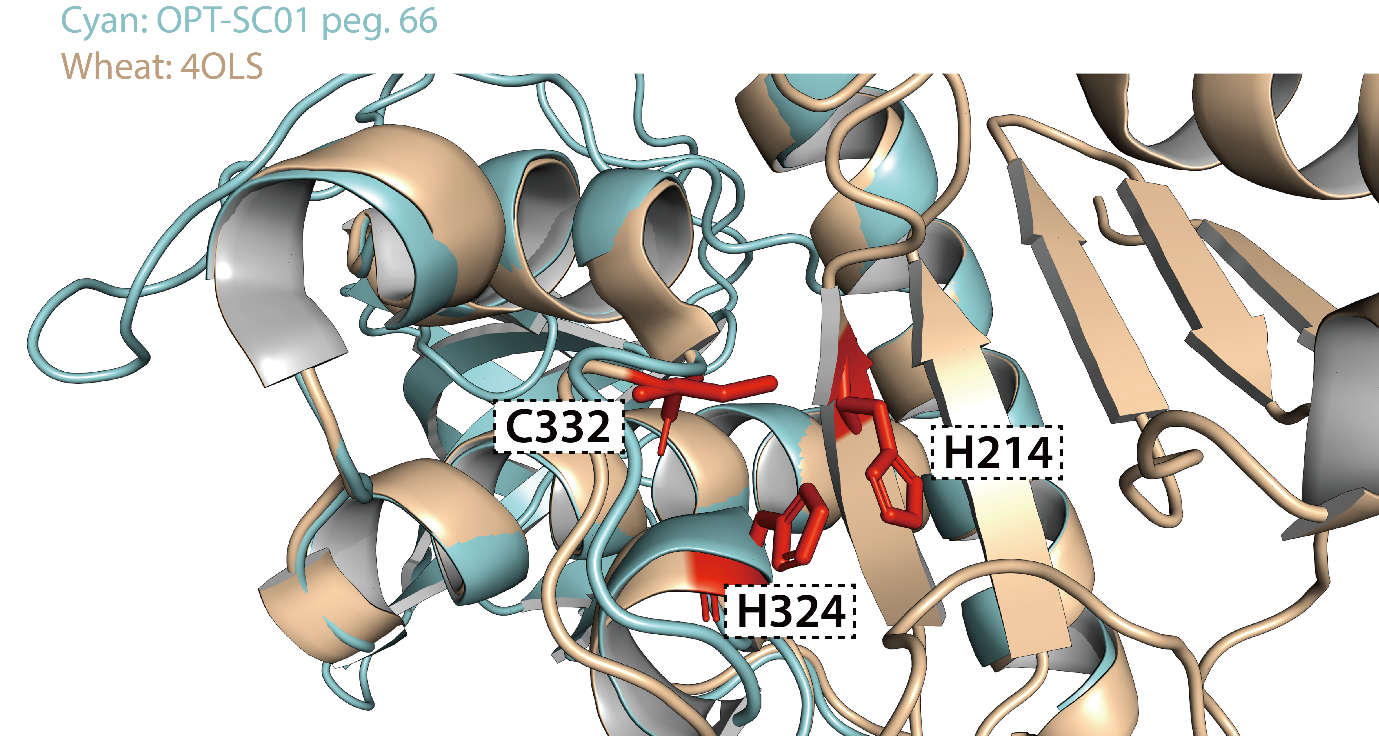


**Figure S4. Structural analysis of the predicted N-acetylmuramoyl-L-alanine amidase domain from OPT-SC01 phage endolysin.**

The predicted structure of the N-acetylmuramoyl-L-alanine amidase domain from OPT-SC01 phage endolysin (cyan) is aligned with a reference amidase structure (4OLS, wheat). This domain is one of multiple domains in the OPT-SC01 endolysin, connected by linkers to other domains including a cell wall binding domain (CBD). Despite incomplete prediction of the overall endolysin structure, the catalytic triad residues (C332, H214, and H324, shown in red) of the amidase domain are conserved.
